# Do Living Arrangements and Social Network Influence the Mental Health Status of Older Adults in Malaysia?

**DOI:** 10.3389/fpubh.2021.624394

**Published:** 2021-05-05

**Authors:** Tengku Aizan Hamid, Hazwan Mat Din, Mohamad Fazdillah Bagat, Rahimah Ibrahim

**Affiliations:** ^1^Malaysian Research Institute on Ageing, Universiti Putra Malaysia, Serdang, Malaysia; ^2^Department of Human Development and Family Studies, Faculty of Human Ecology, Universiti Putra Malaysia, Serdang, Malaysia

**Keywords:** social network, living arrangement, mental health, older adults, interaction effect, Malaysia

## Abstract

Living arrangement has been reported to have a significant influence on several mental health statuses of older adults, but their social network may confound this association. This study is aimed at examining the interactive effect of living arrangements and social network on the mental health status among older adults in Malaysia. A total of 2,188 Malaysian older adults living nationwide were included in this cross-sectional study. Participants were classified into four groups according to their living arrangements (living alone or not living alone) and social network size (assessed using Lubben's Social Network Scale-6). Poor social network was defined as the lowest quartile (fourth quartile) of the score. Mental health statuses, which include flourishing in life, life satisfaction, cognitive functions, loneliness, depression, and perceived stress, were measured. Multiple linear regression models, adjusted for age, gender, education, and comorbidities, revealed that a good social network was significantly associated with an increase on the flourishing scale scores, regardless of living arrangements. Not living alone and having good social network was significantly associated with increased Montreal Cognitive Assessment scores and decreased loneliness scores. This study found that living arrangements are not always a risk factor for the mental health status of older adults. However, it may be confounded by the level of their social networks. The results suggested that the effects of social network may exceed the impact of living arrangements. It is recommended that health professionals pay more attention to the social networks of older Malaysians to harness its benefits in improving their mental health status.

## Introduction

The proportion of older adults in the overall population has been increasing substantially not only in the developed countries but also in many developing nations (1, 2). A recent report conducted by the United Nations (3) estimated that by 2050, one in six people in the world would be over the age of 65, up from 1 to11 in 2019. The increasing numbers of older people come with a challenge to promote and maintain the health system and quality of life (1, 2, 4, 5). A body of literature has documented the role of type of living arrangements and networks in shaping the mental health and life satisfaction of older adults (5–12). Older adults with stronger social support have an increased likelihood of better health and higher increase in mental well-being than the socially disengaged (8, 10). In contrast, older adults who were living in a poor living arrangement (e.g., living alone) or who had a weak social network were suffering from conditions such as higher dementia (13), higher depressive symptom scores (14, 15), higher loneliness level (16, 17), lower general cognitive ability (18), lower levels of vitality (19), and higher mortality risk (20). The existing evidence revealed different aspects of an individual's social relationships that indicated that the structural deficits or abundance of one's network (10) has an effect on mental health outcomes. However, most of the existing studies investigate specific aspects of social relationships and its effect on individuals' mental health like the deficits of social network (15, 21, 22).

Social isolation and poor social networks are considered as a major public health threat (8) among older adults. A social network is the pattern of communication ties of a person, group, or community (23). Social support could also refer to the individuals' connection with others and the community, which include interpersonal interaction and communication, affection and companionship, caring and concern, financial assistance, and acceptance and respect (2, 24). The social network assessments include both the degree of connectedness (like weak and strong ties) and network composition (like family, friends, etc.) (25). Previous studies also stated that the effects of social relationships can be understood through understanding the components of supportive relations that indicate the degree of social integration, the actual support obtained from the network, and the satisfaction with the relationship (26). Prior literature had demonstrated the consequences of poor social network on individuals' quality of life (8, 27), mental health outcomes (9, 28, 29), cognitive decline (30, 31), mortality (20), and psychological well-being (32). Social network yielded various health outcomes including protection against depression and loneliness (16, 17, 20, 28), promotion of higher life satisfaction (8, 9, 33), safeguarding against Alzheimer's disease and dementia (34), improvement in cognitive function (30), and maintenance of general health (26, 29). Previous studies had also revealed various types of social network structures that were based on diverse or restricted social ties (8, 10).

Concomitantly, living arrangements could refer to the type of individual connections that bind the familial and non-familial relationships of a person to the other people they reside with (15). There are various types of living arrangements for older adults, which include living with a spouse, living alone, and living with others (35, 36). The types of living arrangements could also play an essential role in the levels of life satisfaction (2, 36), social support (4), various health outcomes (10, 11, 37), and psychological well-being (38). Living in households consisting of more than one individual is an essential feature for good mental health and recovery process, particularly for older adults (37, 39). Generally, urban communities' living arrangements have transitioned from a co-residence pattern to a more self-independent pattern, affecting the older adults' mental health status (3, 8, 40). However, other studies showed that family proximity was not related to older adults' mental health (41).

Generally, both social networks and living arrangements of older adults could play a critical role in their mental health status (14–17). Several studies have examined the correlation between mental health status and engagements in social activities (13, 15, 18, 20); however, there is a lack of evidence about the interaction effects of living arrangements and social networks on mental health among older adults (14, 20). In the Malaysian context, the number of individuals aged 65 years and above is estimated to be 6.7% of the population and is expected to increase to 15% over the next 10 years (42).

It is a cultural norm for older adults to co-reside with family members in Malaysia (2). Noted that 71% of older adults in Peninsular Malaysia were living with adult children, 16% were living with spouse only, and 9% were living alone. The recent 5th Malaysia Population and Family Life Survey documented over 70% of older Malaysians who were living with family members and others in their household, about 21% were living with spouse only, and 9% were living alone (43). Using the national household income and expenditure survey, (44) also recorded that over 60% of the elderly were living in co-residency with adult children, 18% were living in spouse-only arrangements, and 6% were living alone. Nevertheless, (44) noted that living arrangements were influenced by age, gender, ethnicity, location of residence, and marital status. Male elderly who lived in rural areas are more likely to live alone when becoming older, but the Chinese elderly are more likely to co-reside with an adult child. In addition, married elderly, were more likely to live in spouse-only living arrangements. Thus, far, only (2), had studied the relationship between living arrangements and life satisfaction among older Malaysians living in Peninsular Malaysia. They reported that older adults living in spouse-only arrangements and co-residency with adult children recorded higher life satisfaction levels compared with those living alone. Living arrangements showed direct and indirect effects on life satisfaction through the support function. This implied that supportive relationships and interactions between members in the living unit promote better evaluation of their life satisfaction.

Nonetheless, scientific evidence of social network and mental health among Malaysian older adults are few. Besides, the interaction effect of social network types and living arrangement types on the mental health of older adults have not been extensively investigated. Considering the lack of network studies in the Malaysian context, the purpose of the current study was to examine the interaction effect of living arrangements and social network on the mental health status among older adults in Malaysia.

**Figure 1:**
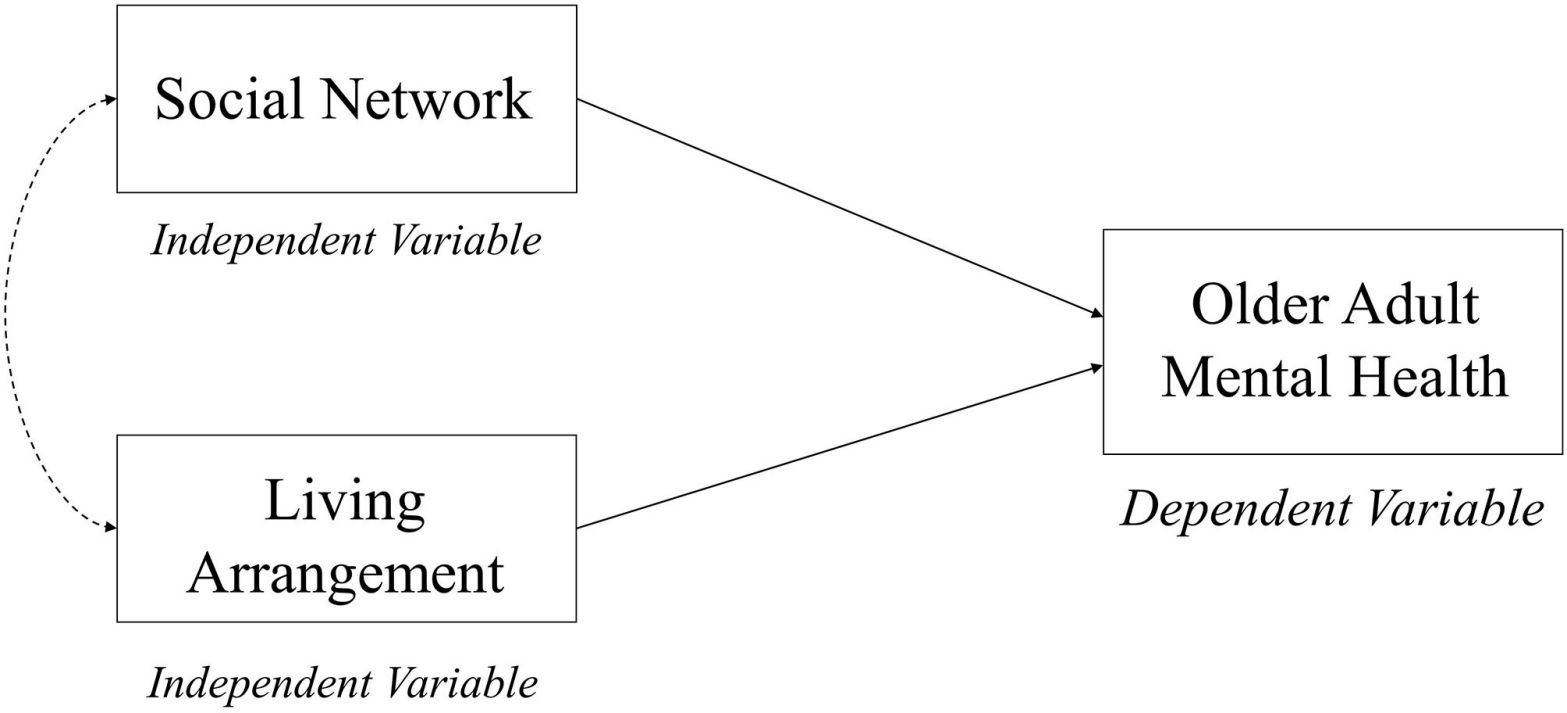
The conceptual framework of the interaction model.

The Social Convoy model was considered as the theoretical basis for this study because it discusses the nature of social relationships and their influence on health and well-being (45). This model was first developed by (45) to capture the social relations and social ties across the life span. They described the social relations as a convoy, where persons are surrounded by supportive people who move with them throughout their lives (45–47). These relationships may vary in terms of function (affect, aid, or affirmation exchanges), quality (positive or negative), and structure (composition, frequency, size or arrangements, and geographic proximity), and these have significant impacts on their physical and mental health (45–47). The dimensions of convoys could be influenced by characteristics (e.g., age, gender, and occupation) and situational factors (e.g., demands and resource) (45, 47, 48). The convoy model also contributed to the study of interpersonal relationships and support in adulthood and aging (47). Consequently, the concept of Social Convoy has increasingly been included in the recent gerontological research to consider the current circumstances and possible predictions of the health, well-being, and lives of older adults (48). Furthermore, the definitions of social networks highlighted the role of social support and living arrangements on the psychological well-being of older adults (9, 10, 28). Based on the theoretical evidence, a conceptual framework of the current study was developed to determine the interaction effects of living arrangements and social network on the mental health status among older adults living in Malaysia (see Figure 1).

## Methods

### Participants and Study Design

The data for the study consisting of 2,322 community-dwelling older adults were obtained from a nationally representative population-based survey entitled, Identifying Psychosocial and Identifying Economic Risk Factor of Cognitive Impairment among Elderly, conducted in Peninsular Malaysia. This study is a sub-project under a longitudinal study on a neuro protective model for healthy longevity (LRGS TUA). The details of the methodology have previously been published elsewhere (49). A multistage random sampling of Malaysian older adults from four states in Malaysia, i.e., Johor, Selangor, Kelantan, and Perak, were performed, and outcome measures were followed at 18 and 36 months. Data collection was conducted using a face-to-face interview by trained enumerators in a nearby community hall from May 2012 to February 2013, with a response rate of 90%. For this paper, only the first wave data were used.

### Ethical Consideration

This study was approved by the Medical Research and Ethics Committee of Universiti Kebangsaan Malaysia. Oral informed consent was obtained from all respondents who agreed to participate in the study.

### Measurement Tools

#### Living Arrangements and Social Network

Living arrangements of respondents were determined by asking the question, “Are you living alone or with others?” with the answer options of, “Alone,” “With a spouse,” “With children,” “With relatives,” or “With others.” Those who answered, “Alone,” were assigned to the “Living alone group,” while other responses were assigned to the “Not living alone group.”

Social network was measured using the abbreviated version of the Lubben Social Network Scale (LSNS-6) (50), which consists of six questions assessing the size of three different aspects of social network such as active social network, perceived support network, and perceived confidant network. Those three aspects are attributed to family ties and a parallel set is attributable to friendship ties. Each question is scored from 0 to 5 with a maximum score of 30. A higher score indicates a larger social network.

#### Flourishing Scale

The Flourishing Scale is an eight-item questionnaire Likert scale that measures self-perceived success in areas of relationship, self-esteem, purpose, and optimism (51). Respondents can choose an answer using a Likert scale ranging from 1 (Strongly disagree) to 7 (strongly agree). Total scores are calculated, and the sum of all item score ranges from 8 to 56; higher scores indicate greater flourishing in life. The scale is reported to have excellent reliability among the Malaysian population (52).

#### Satisfaction With Life Scale

The Satisfaction with Life Scale (SWLS) (33) is a five-item questionnaire that measures global cognitive judgments of participants' life satisfaction, without measuring Neither positive or negative affect. Participants need to choose an answer from a scale that ranges from 1 (Disagree) to 3 (Agree). Total scores were calculated by summing up all items answered (ranges from 5 to 15), whereby a higher score indicates greater satisfaction. A previous study had reported SWLS as a valid and reliable measure of life satisfaction among Malaysian samples (53).

#### Montreal Cognitive Assessment

Montreal Cognitive Assessment (MOCA) is a screening tool to measure cognitive impairments. The scores range from 0 to 30; a higher score indicates better cognitive function. MOCA was reported as a valid and reliable scale to measure cognitive impairments among Malaysian older adults (54).

#### Loneliness Scale

The three-item Loneliness Scale is a questionnaire developed from the Revised UCLA Loneliness Scale. Each question is rated on a three-point scale (1 = Hardly Ever; 2 = Some of the Time; 3 = Often). The scale asks about how often the respondent feels that they lack companionship, feels left out, and feels isolated from others. All items are summed up to give a total score (ranges from 3 to 9). A higher score indicates greater loneliness. A previous study indicated that total reliability, concurrent and discriminant validity of the scales were satisfactory (55).

#### Geriatric Depression Scale

The Geriatric Depression Scale (GDS) (49) is a 15-item questionnaire used to screen for depression, specifically developed for uses with older adults. The items are rated on a binary response (Yes/No). The GDS total score in this study was calculated based on the sum of all item response. A higher score indicates greater depression. The scale reliability and validity have been established among the Malaysian older adult population (56).

#### Perceived Stress Scale

The Perceived Stress Scale (PSS) was developed to measure how controllable and unpredictable people viewed their lives, which is an essential component of the experience of stress (57). This study used the short form four-item PSS rated from 0 (Never) to 4 (Very often). Two items were negatively stated, and another two were positively stated. The total scores ranged from 0 to 16. A higher score indicates greater stress. A validation study reported that PSS has a satisfactory reliability and validity among the sample of older adults (58).

### Data Analysis

To examine the interaction effects of living arrangements and social network, the LSNS-6 scores among all respondents were first divided into quartiles, where the lowest quartile (fourth quartile) was defined as the poor social network group, while the first to third quartiles are referred to as good social network groups. The respondents were then categorized into four groups as follows: Group 1, living alone and poor social network; Group 2, living alone and good social network; Group 3, not living alone and poor social network; Group 4, not living alone and good social network.

The descriptive statistics of the study variables among the four groups were examined using the Chi-square test for the categorical variables and *t*-tests for continuous variables. To assess the interaction effects of living arrangements and social network on the continuous mental health variables, multiple linear regression analyses were performed on Flourishing in Life Scale, SWLS, MOCA, Loneliness Scale, GDS, and PSS. Each regression models were adjusted for confounders: age, gender, education level, and comorbidities. All statistical analyses were performed using the IBM SPSS v22.0 (SPSS Inc. Chicago, IL).

## Results

A total of 2,188 respondents were included in this study (mean age 68.95 ± 6.17, with 51.7% women and 48.3% men). Table 1 presents the characteristics of respondents stratified by the combination of living arrangements and quality of their social network. Approximately, 67.2% of the respondents were not living alone and had good social network. Only 10.1% of the respondents were living alone, and 3.2% had poor social network, while 6.9% had good social network. Majority of women were living alone, regardless of having poor (70.4%) or good social network (72.2%). Respondents not living alone recorded higher educational achievements than respondents living alone, regardless of their social network quality. The mean score of the Flourishing in Life Scale score was almost equal in Group 2 (50.62%) and Group 4 (50.93%) respondents, respectively, and the differences were significant. Group 4 respondents recorded the highest SWLS (8.22). In addition, Group 4 also recorded the highest MOCA mean score (19.08). As expected, Group 1 respondents exhibited highest Loneliness (3.58) and GDS (7.25) scores. Highest mean score for PSS (3.19) was shown in Group 2, while Lubben score was highest in Group 4 (3.19).

**Table 1:** Characteristics of respondents stratified by the combination of living arrangement and quality of social network.

**Characteristics**	**LA and poor SN (Group 1)**	**LA and good SN (Group 2)**	**Not LA and poor SN (Group 3)**	**Not LA and good SN (Group 4)**	***P***
	***n* = 71 (3.2%)**	***n* = 151 (6.9%)**	***n* = 495 (22.6%)**	***n* = 1,471 (67.2%)**	
Age, mean (SD)	70.52 (5.82)	70.87 (6.17)	68.91 (5.93)	68.69 (6.23)	** <0.001**
Women, *n* (%)	50 (70.4)	109 (72.2)	263 (53.1)	710 (48.3)	** <0.001**
Men, *n* (%)	21 (29.6)	42 (27.8)	232 (46.9)	761 (51.7)	
Education, mean (SD)	3.86 (3.91)	4.08 (3.85)	5.08 (3.93)	5.45 (3.98)	** <0.001**
Hypertension, *n* (%)	31 (43.7)	69 (45.7)	232 (46.9)	670 (45.5)	0.941
Diabetes mellitus, *n* (%)	21 (29.6)	36 (23.8)	106 (21.4)	358 (24.3)	0.377
Arthritis, *n* (%)	6 (8.5)	19 (12.6)	45 (9.1)	163 (11.1)	0.474
Heart disease, *n* (%)	5 (7.0)	15 (9.9)	38 (7.7)	137 (9.3)	0.632
Flourishing, mean (SD)	48.35(7.07)	50.62 (6.46)	48.38 (8.71)	50.93 (5.80)	** <0.001**
SWLS, mean (SD)	7.70 (2.71)	8.10 (2.52)	8.06 (2.52)	8.22 (2.34)	0.196
MOCA, mean (SD)	16.13 (5.46)	17.27 (5.68)	18.52 (5.60)	19.08 (5.79)	** <0.001**
Loneliness, mean (SD)	3.58 (1.38)	3.36 (1.06)	3.38 (1.09)	3.20 (0.77)	** <0.001**
GDS, mean (SD)	7.25 (2.03)	6.99 (1.64)	6.93 (1.62)	6.99 (1.64)	0.297
PSS, mean (SD)	3.10(3.10)	3.19 (3.06)	3.18 (3.22)	3.14 (2.99)	0.994
Lubben score, mean (SD)	4.03 (2.31)	14.93 (5.06)	4.13 (2.21)	15.37 (5.23)	** <0.001**

*LA, Living alone; SN, Social network; SWLS, Satisfaction with life scale; MOCA, Montreal cognitive assessment; GDS, geriatric depression scale; PSS, perceived stress scale*.

The Chi-square tests and ANOVA tests showed that age, gender, and education level were among the significant socio demographic variables between the four groups. Assessing the mental health variables revealed that Flourishing Scales, MOCA, and Loneliness Scale scores were significantly different across the four groups. Mean scores for Flourishing Scale and MOCA were lowest among those living alone and having poor social network (Group 1). In addition, Group 1 also showed higher scores on the Loneliness scale.

Table 2 presents the results of multiple linear regression analysis to examine the interaction effects of living arrangements and social network on the mental health variables. The regression models were adjusted for potential confounders: age, gender, education level, and comorbidities. The results showed that Group 4 (Not LA and good SN) was associated with Flourishing Scale scores and MOCA scores. Group 2 (LA and good SN) was significantly associated with high Flourishing Scale scores. Group 4 was also significantly associated with low Loneliness Scale scores. There was no significant association between the four groups and SWLS, GDS, and PSS scores.

**Table 2:** Result of multiple linear regression for mental health variables.

**Status**	**Flourishing Scale**		**SWLS**		**MOCA**	
	**β (95% CI)**	***P***	**β (95% CI)**	***P***	**β (95% CI)**	***P***
Group 1	Reference		Reference		Reference	
Group 2	**2.30 (0.43, 4.17)**	**0.016**	0.38 (−0.27, 1.03)	0.254	1.14 (−0.16, 2.44)	0.084
Group 3	−0.30 (−1.95, 1.36)	0.722	0.36 (−0.21, 0.94)	0.215	1.07 (−0.09, 2.22)	0.070
Group 4	**2.17 (0.59, 3.77)**	**0.008**	0.54 (−0.01, 1.09)	0.054	**1.28 (0.18, 2.39)**	**0.023**
**Status**	**Loneliness Scale**		**GDS**		**PSS**	
	**β** **(95% CI)**	***P***	**β** **(95% CI)**	***P***	**β** **(95% CI)**	***P***
Group1	Reference		Reference		Reference	
Group 2	−0.20 (−0.45, 0.05)	0.122	−0.22 (−0.67, 0.24)	0.351	0.10 (−0.76, 0.95)	0.824
Group 3	−0.14 (−0.36, 0.09)	0.225	−0.18 (−0.58, 0.23)	0.393	0.20 (−0.56, 0.95)	0.609
Group 4	**−0.33 (−0.55**, **−0.12)**	**0.002**	−0.19 (−0.58, 0.20)	0.333	0.19 (−0.54, 0.91)	0.608

*Bold values are statistically significant at P < 0.05*.

*SWLS, Satisfaction with life scale; MOCA, montreal cognitive assessment; GDS, geriatric depression scale; PSS, perceived stress scale; β, regression coefficient; 95%, 95% confidence interval*.

## Discussion

In this study, we have examined the interaction effects of living arrangements and social network on the mental health status of older adults in Malaysia. The study found that the interaction between living arrangements and social network has a significant impact on psychological well-being (Flourishing in Life Scale), cognitive function (MOCA), and loneliness. The current findings reinforce the results of previous studies in which the restricted social network of older adults was associated with more health and mental health risks than other types of social networks (10, 59, 60). More specifically, the results of the multiple regression analysis showed that older adults who have good social network, regardless of living arrangements, recorded higher psychological well-being than those who live alone and have a low social network. This is in line with the results of a quantitative study from China, which found that older adults who have a certain level of social interaction had lower odds of mental health problems (1).

Furthermore, the current study revealed that Malaysian older adults who did not live alone and had good social network reported higher cognitive functions and lower loneliness level than those who are living alone. Social network and social support seem to contribute to a better global cognitive function and episodic memory of older adults (30). Additionally, evidence found multiple associations of low social network, loneliness, and well-being of the Asian older adults (27). The finding of this study also observed that living arrangement is not always a risk factor for the mental health status among older Malaysians. Similarly, an empirical study from the Eastern United States found that living arrangement was not related to elderly mental well-being (41). However, another quantitative study from Malaysia conducted by (2) found that the living arrangements, directly and indirectly, play an essential role in predicting life satisfaction for older adults. The difference in results may be due to the different operationalization of living arrangements used in the current study, where the levels of the social network were also imputed.

On the other hand, the results of multiple regression analysis of the current study showed no significant associations among various types of interactions between social network and living arrangements with the scales of satisfaction with life, geriatric depression, and perceived stress. Our results contradicted previous quantitative studies regarding the role of diverse social ties of older adults in protecting against depression (9), achieving a higher life satisfaction (2), and reducing stress level (61). Previous studies did not include the interaction effects of social networking and living arrangements on the mental health well-being of older adults like we did in this paper. Furthermore, the current study showed that the demographic characteristics (such as age, gender, and education level) were significantly associated with diverse types of combinations for social network and living arrangements. This finding is consistent with studies conducted by (44, 62), where they corroborated that living arrangements were correlated with different socio demographic characteristics of older adults.

Our study showed that when there is good quality of social network, Malaysian older adults from different living arrangements tend to have higher mental health well-being. Our findings also supports the (45) Social Convoy assertion that strong social network will translate into support network, which can be utilized when needed. The reassurance of support through the investment in long-term social relations buffers the impact of poor living arrangements such as living alone (32). Also suggested that social network is an important resource in the older adults' life as it improves their psychological well-being.

The findings of this study add new knowledge to the emerging literature on social network, living arrangements, and the mental health status of older adults. Our results indicated that the combined effects of living arrangements and strong social support network influence the mental health status of older Malaysians. Therefore, future intervention programs to improve the mental health status of the elderly, particularly in Malaysia and Southeast Asia, would need to consider the characteristics of the social network in the design of the intervention. It would be useful for the older adults and those intimately associated with them in understanding the relationship between socialization and living arrangements with the older adults' mental health status. Furthermore, the findings of this study indicate the need for attention to the mental health of the elderly who are living alone and having poor social support.

Nevertheless, the study has some limitations. First, even though the sample of the present study is large, the generalizability of the findings is limited as a case-centered approach was used to obtain the data, and optimal group is specific to the selected sample. Second, the analysis only involves first-wave respondents; therefore, only associational relationships were established, and causality cannot be ascertained. Third, the data were self-reported and may have inherent bias.

## Data Availability Statement

The raw data supporting the conclusions of this article will be made available by the authors.

## Ethics Statement

The studies involving human participants were reviewed and approved by Medical Research and Ethics Committee of Universiti Kebangsaan Malaysia. The patients/participants provided their written informed consent to participate in this study.

## Author Contributions

The study concept was developed by TH. The manuscript was drafted by TH, HD, and MB. Data analysis was performed by HD. Data collection and management were completed by MB and TH. The manuscript was critically revised by TH, HD, MB, and RI. All authors contributed to the article and approved the submitted version.

## Conflict of Interest

The authors declare that the research was conducted in the absence of any commercial or financial relationships that could be construed as a potential conflict of interest.

## References

[1] Sun X Lucas H Meng Q Zhang Y. Associations between living arrangements and health-related quality of life of urban elderly people: a study from China. Quality Life Res. (2011) 20:359–69. 10.1007/s11136-010-9752-z 20878548

[2] Kooshiar H Yahaya N Hamid TA Abu Samah A Sedaghat Jou V. Living arrangement and life satisfaction in older Malaysians: the mediating role of social support function. PLoS ONE. (2012) 7:e43125. 10.1371/journal.pone.0043125 22912806 PMC3422218

[3] Feng Z Falkingham J Liu X Vlachantoni A. Changes in living arrangements and mortality among older people in China. SSM Popul Health. (2017) 3:9–19. 10.1016/j.ssmph.2016.11.009 29349200 PMC5768996

[4] Lewis JS. Housing and social support needs of elderly persons: A needs assessment in an independent living facility. Eval Pro Plan. (1997) 20:269–77. 10.1016/S0149-7189(97)00005-0

[5] Westerhof GJ Keyes CL. Mental illness and mental health: the two continua model across the lifespan. J Adult Dev. (2010) 17:110–9. 10.1007/s10804-009-9082-y 20502508 PMC2866965

[6] Zunzunegui MV Koné A Johri M Béland F Wolfson C Bergman H. Social networks and self-rated health in two french-speaking canadian community dwelling populations over 65. Soc Sci Med. (2004) 58:2069–81. 10.1016/j.socscimed.2003.08.005 15020020

[7] Yoo JA Zippay A. Social networks among lower income Korean elderly immigrants in the U.S. J Aging Stud. (2012) 26:368–76. 10.1016/j.jaging.2012.03.005

[8] Park S Smith J Dunkle RE. Social network types and well-being among South Korean older adults. Aging Men Health. (2014) 18:72–80. 10.1080/13607863.2013.801064 23741987

[9] Singh L Singh PK Arokiasamy P. Social network and mental health among older adults in rural uttar pradesh, India: a cross-sectional study. J Cross Cult Gerontol. (2016) 31:173–92. 10.1007/s10823-016-9286-0 26879450

[10] Park NS Jang Y Lee BS Chiriboga DA Chang S Kim SY. Associations of a social network typology with physical and mental health risks among older adults in South Korea. Aging Men Health. (2018) 22:631–8. 10.1080/13607863.2017.1286456 28290722

[11] Oshio T Kan M. Which is riskier for mental health, living alone or not participating in any social activity? Evidence from a population-based eleven-year survey in Japan. Soc Sci Med. (2019) 233:57–63. 10.1016/j.socscimed.2019.05.049 31176058

[12] Wu F Sheng Y. Social support network, social support, self-efficacy, health-promoting behavior and healthy aging among older adults: a pathway analysis. Arch Gerontol Geriatr. (2019) 85:103934. 10.1016/j.archger.2019.103934 31466024

[13] Fratiglioni L Wang H-X Ericsson K Maytan M Winblad B. Influence of social network on occurrence of dementia: a community-based longitudinal study. Lancet. (2000) 355:1315–9. 10.1016/S0140-6736(00)02113-9 10776744

[14] Chou KL Ho AHY Chi I. Living alone and depression in Chinese older adults. Aging Men Health. (2006) 10:583–91. 10.1080/13607860600641150 17050087

[15] Chan A Malhotra C Malhotra R Østbye T. Living arrangements, social networks and depressive symptoms among older men and women in Singapore. Int J Geriatr Psychiatry. (2011) 26:630–9. 10.1002/gps.2574 20677171

[16] Kuroda A Tanaka T Hirano H Ohara Y Kikutani T Furuya H. Eating alone as social disengagement is strongly associated with depressive symptoms in Japanese community-dwelling older adults. J Am Med Dir Asso. (2015) 16:578–85. 10.1016/j.jamda.2015.01.078 25687929

[17] Shorey S Chan V. The experiences and needs of Asian older adults who are socially isolated and lonely: a qualitative systematic review. Arch Gerontol Geriatr. (2021) 92:104254. 10.1016/j.archger.2020.104254 32957019

[18] Gow AJ Corley J Starr JM Deary IJ. Which social network or support factors are associated with cognitive abilities in old age? Gerontology. (2013) 59:454–63. 10.1159/000351265 23711796

[19] Michael YL Berkman LF Colditz GA Kawachi I. Living arrangements, social integration, and change in functional health status. Am J Epidemiol. (2001) 153:123–31. 10.1093/aje/153.2.123 11159156

[20] Kauppi M Kawachi I Batty GD Oksanen T Elovainio M Pentti J. Characteristics of social networks and mortality risk: evidence from 2 prospective cohort studies. Am J Epidemiol. (2017) 187:746–53. 10.1093/aje/kwx301 29020140

[21] Holwerda TJ Beekman AT Deeg DJ Stek ML van Tilburg TG Visser PJ. Increased risk of mortality associated with social isolation in older men: only when feeling lonely? Results from the Amsterdam Study of the Elderly (AMSTEL). Psychol Med. (2012) 42:843–53. 10.1017/S0033291711001772 21896239

[22] Sakurai R Kawai H Suzuki H Kim H Watanabe Y Hirano H. Poor social network, not living alone, is associated with incidence of adverse health outcomes in older adults. J Am Med Dir Asso. (2019) 20:1438–43. 10.1016/j.jamda.2019.02.021 31000349

[23] Weenig M. Social networks. In: Spielberger C editors. Encyclopedia of Applied Psychology. New York, NY: Elsevier (2004). p. 421–6.

[24] Antonucci T Akiyama H Sherman A. Social networks, support, and integration. Encyclo Gerontol. (2007) 531–41. 10.1016/B0-12-370870-2/00175-X

[25] Litwin H Shiovitz-Ezra S. The association of background and network type among older Americans: is “who you are” related to “who you are with?” *Res Aging*. (2011) 33:735–59. 10.1177/0164027511409441

[26] Li T Zhang Y. Social network types and the health of older adults: Exploring reciprocal associations. Soc Sci Med. (2015) 130:59–68. 10.1016/j.socscimed.2015.02.007 25681715

[27] Shin SH Sok SR. A comparison of the factors influencing life satisfaction between Korean older people living with family and living alone. Int Nur Rev. (2012) 59:252–8. 10.1111/j.1466-7657.2011.00946.x 22591098

[28] Litwin H. The association between social network relationships and depressive symptoms among older Americans: what matters most? Int Psychoger. (2011) 23:930. 10.1017/S1041610211000251 21356159

[29] Lam J Bolano D. Social and productive activities and health among partnered older adults: a couple-level analysis. Soc Sci Med. (2019) 229:126–33. 10.1016/j.socscimed.2018.04.016 29691088

[30] Kelly ME Duff H Kelly S McHugh Power JE Brennan S Lawlor BA. The impact of social activities, social networks, social support and social relationships on the cognitive functioning of healthy older adults: a systematic review. Syst Rev. (2017) 6:259. 10.1186/s13643-017-0632-2 29258596 PMC5735742

[31] Micheli K Ratsika N Vozikaki M Chlouverakis G Philalithis A. Family ties and functional limitation in the elderly: results from the survey of health ageing and retirement in Europe (SHARE). Archiv Gerontol Geriatr. (2018) 78:23–29. 10.1016/j.archger.2018.05.023 29883806

[32] Momtaz YA Hamid TA Yahaya N Ibrahim R. Widowhood and psychological well-being among older Malaysians: mediating effect of social network. Ind J Soc Work. (2009) 70:375–90.

[33] Diener E Emmons RA Larsen RJ Griffin S. The satisfaction with life scale. J Pers Assess. (1985) 49:71–75. 10.1207/s15327752jpa4901_13 16367493

[34] Fratiglioni L Paillard-Borg S Winblad B. An active and socially integrated lifestyle in late life might protect against dementia. Lancet Neurol. (2004) 3:343–53. 10.1016/S1474-4422(04)00767-7 15157849

[35] Kim G Jang Y Chiriboga DA. Personal views about aging among Korean American older adults: the role of physical health, social network, and acculturation. J Cross Cult Gerontol. (2012) 27:139–48. 10.1007/s10823-012-9165-2 22581472

[36] Kim HJ Hong S Kim M. Living arrangement, social connectedness, and life satisfaction among Korean older adults with physical disabilities: the results from the National Survey on persons with disabilities. J Dev Phys Dis. (2015) 27:307–21. 10.1007/s10882-014-9418-9

[37] Teerawichitchainan B Pothisiri W Long GT. How do living arrangements and intergenerational support matter for psychological health of elderly parents? Evidence from myanmar, vietnam, and thailand. Soc Sci Med. (2015) 136–137:106–16. 10.1016/j.socscimed.2015.05.019 25993521

[38] Yamada K Teerawichitchainan B. Living arrangements and psychological well-being of the older adults after the economic transition in Vietnam. J Gerontol Series B. (2015) 70:957–68. 10.1093/geronb/gbv059 26307484

[39] Pernice-Duca F. Family network support and mental health recovery. J Mar Family Ther. (2010) 36:13–27. 10.1111/j.1752-0606.2009.00182.x 20074121

[40] Ye M Chen Y. The influence of domestic living arrangement and neighborhood identity on mental health among urban Chinese elders. Aging Mental Health. (2014) 18:40–50. 10.1080/13607863.2013.837142 24044640

[41] McCulloch BJ. The relationship of family proximity and social support to the mental health of older rural adults: the appalachian context. J Aging Stud. (1995) 9:65–81. 10.1016/0890-4065(95)90026-8

[42] Mat Din H Nor Akahbar SA Ibrahim R. The association between depression and sexual satisfaction among Malay elderly in Malaysia. Heliyon. (2019) 5:e01940. 10.1016/j.heliyon.2019.e01940 31338454 PMC6579850

[43] Mahmud A Jaffar W Hashim W Mohammad AH Ishak I Sapri M. Report on Key Findings Fifth Malaysian Population and Family Survey (MPFS-5) 2014. (2016). Available online at: http://familyrepository.lppkn.gov.my/659/ (accessed January 15, 2021).

[44] Mohd S Senadjki A Mansor N. Living arrangements of elderly: evidence from household income expenditure survey. J Popul Ageing. (2017) 10:323–42. 10.1007/s12062-016-9165-z

[45] Kahn R Antonucci T. Convoys over the life course: attachment, roles and social support. Life Span Dev Behav. (1980) 3:253–86.

[46] House JS Robbins C Metzner HL. The association of social relationships and activities with mortality: prospective evidence from the tecumseh community health study. Am J Epidemiol. (1982) 116:123–40. 10.1093/oxfordjournals.aje.a113387 7102648

[47] Antonucci TC Ajrouch KJ Birditt KS. The Convoy Model: explaining social relations from a multidisciplinary perspective. Gerontologist. (2013) 54:82–92. 10.1093/geront/gnt118 24142914 PMC3894851

[48] Fuller-Iglesias H Smith J Antonucci T. Theoretical perspectives on life span and life course development. Annual Rev Gerontol Geriatr. (2010) 29:3–25. 10.1891/0198-8794.29.3

[49] Shahar S Omar A Vanoh D Hamid TA Mukari SZMS Din NC. Approaches in methodology for population-based longitudinal study on neuroprotective model for healthy longevity (TUA) among malaysian older adults. Aging Clin Exp Res. (2016) 28:1089–104. 10.1007/s40520-015-0511-4 26670602

[50] Lubben J Blozik E Gillmann G Iliffe S von Renteln Kruse W Beck JC. Performance of an abbreviated version of the Lubben Social Network Scale among three European community-dwelling older adult populations. Gerontologist. (2006) 46:503–13. 10.1093/geront/46.4.503 16921004

[51] Diener E Wirtz D Tov W Kim-Prieto C Choi DW Oishi S. New well-being measures: short scales to assess flourishing and positive and negative feelings. Soc Ind Res. (2010) 97:143–56. 10.1007/s11205-009-9493-y

[52] Momtaz YA Hamid TA Haron SA Bagat MF. Flourishing in later life. Arch Gerontol Geriatr. (2016) 63:85–91. 10.1016/j.archger.2015.11.001 26627531

[53] Swami V Chamorro-Premuzic. Psychometric evaluation of the malay satisfaction with life scale. Soc Ind Res. (2008) 92:25. 10.1007/s11205-008-9295-7

[54] Din NC Shahar S Zulkifli BH Razali R Vyrn CA Omar A. Validation and optimal cut-off scores of the bahasa Malaysia version of the montreal cognitive assessment (MoCA-BM) for mild cognitive impairment among community dwelling older adults in Malaysia. Sains Malaysiana. (2016) 45:1337–43.

[55] Hughes ME Waite LJ Hawkley LC Cacioppo JT. A short scale for measuring loneliness in large surveys: results from two population-based studies. Res Aging. (2004) 26:655–72. 10.1177/0164027504268574 18504506 PMC2394670

[56] Ewe E Che Ismail H. Validation of malay version of geriatric depression scale among elderly inpatients. Age. (2004) 17:65–64.

[57] Cohen S Kamarck T Mermelstein R. A global measure of perceived stress. J Health Soc Behav. (1983) 24:385–96. 10.2307/2136404 6668417

[58] Ezzati A Jiang J Katz MJ Sliwinski MJ Zimmerman ME Lipton RB. Validation of the perceived stress scale in a community sample of older adults. Int J Geriatr Psychiatry. (2014) 29:645–52. 10.1002/gps.4049 24302253 PMC4013212

[59] Ren Q Treiman DJ. Living arrangements of the elderly in China and consequences for their emotional well-being. Chin Soc Rev. (2015) 47:255–86. 10.1080/21620555.2015.1032162

[60] Tang D Lin Z Chen F. Moving beyond living arrangements: the role of family and friendship ties in promoting mental health for urban and rural older adults in China. Aging Mental Health. (2020) 24:1523–32. 10.1080/13607863.2019.1602589 30977378

[61] Ellwardt L Wittek RPM Hawkley LC Cacioppo JT. Social network characteristics and their associations with stress in older adults: closure and balance in a population-based sample. J Gerontol Series B. (2019) 75:1573–84. 10.1093/geronb/gbz035 30888040 PMC7424276

[62] Sereny MD Gu D. Living arrangement concordance and its association with self-rated health among institutionalized and community-residing older adults in China. J Cross Cult Gerontol. (2011) 26:239–59. 10.1007/s10823-011-9145-y 21484315

